# Evaluation of the Behavior of Phenolic Compounds and Steviol Glycosides of Sonicated Strawberry Juice Sweetened with Stevia (*Stevia rebaudiana* Bertoni)

**DOI:** 10.3390/molecules24071202

**Published:** 2019-03-27

**Authors:** Jana Šic Žlabur, Nadica Dobričević, Mladen Brnčić, Francisco J. Barba, Jose M. Lorenzo, Daniel Franco, Atanas G. Atanasov, Sandra Voća, Suzana Rimac Brnčić

**Affiliations:** 1 Faculty of Agriculture, University of Zagreb, Svetošimunska cesta 25, 10000 Zagreb, Croatia; jszlabur@agr.hr (J.Š.Ž.); ndobricevic@agr.hr (N.D.); 2 Faculty of Food Technology and Biotechnology, University of Zagreb, Pierottijeva 6, 10000 Zagreb, Croatia; srimac@pbf.hr; 3 Nutrition and Food Science Area, Preventive Medicine and Public Health, Food Science, Toxicology and Forensic Medicine Department, Faculty of Pharmacy, Universitat de València, Avda. Vicent Andrés Estellés, s/n, 46100 Burjassot, València, Spain; 4 Centro Tecnológico de la Carne de Galicia, Avda. Galicia No. 4, Parque Tecnológico de Galicia, San Cibrao das Viñas, 32900 Ourense, Spain; jmlorenzo@ceteca.net (J.M.L.); danielfranco@ceteca.net (D.F.); 5 Institute of Genetics and Animal Breeding of the Polish Academy of Sciences, 05-552 Jastrzebiec, Poland; atanas.atanasov@univie.ac.at; 6 Department of Pharmacognosy, University of Vienna, Althanstrasse 9, 1090 Vienna, Austria

**Keywords:** green stevia powder, strawberry juice, antioxidant compounds, steviol glycosides, sonication

## Abstract

In this study, the influence of stevia addition and sonication processing parameters on the phenolic content and profile as well as the steviol glycosides of strawberry juice-based samples was investigated. For this purpose, three matrices—control samples of strawberry juices without green stevia powder (CS), strawberry juices with green stevia powder (JGSP), and sonicated juices with green stevia powder (SJGSP)—were prepared. For sonication purposes, different conditions regarding probe diameters (7 mm and 22 mm), amplitudes (50%, 75%, and 100%), and time (15 min, 20 min, and 25 min) were tested. The results that were obtained upon the measurement of the total phenolic content, total flavonoids, steviol glycosides, and antioxidant capacity showed significant differences according to the matrices evaluated, obtaining overall higher values in the samples with stevia added. Moreover, when sonication was evaluated, it was found that a higher amplitude (100%), a larger probe diameter (22 mm), and a longer sonication period (25 min) led to higher values. Flavones such as luteolin and apigenin were identified and quantified in JGSP and SJGSP, while they were not found in CS. Besides these phenolic compounds, kaempferol, quercetin, pyrogallic acid, 4-methylcatechol, and 4-methoxybenzoic acid were also identified and quantified. Similarly to the total phenolic compounds, total flavonoids, and total antioxidant capacity, an increased amount of these compounds was found in SJGSP, especially after using the most intense sonication conditions. Therefore, the use of sonication together with stevia added could be a useful tool to preserve strawberry juices, increasing at the same time the sweetness and the antioxidant value of the beverages.

## 1. Introduction

Green stevia powder (GSP) is a rich source of various nutrients and phytochemicals such as polyphenols [1,2] with strong antioxidant activity [3,4,5]. In addition, it is a source of non-caloric sweeteners such as stevioside, steviolbioside, rebaudioside (A, B, C, D, E, F), dulcoside A, and rubusoside [6] from natural origin, which can be used in order to replace sugars in food products. Also, some authors in their research provide new phenol and polyphenol compounds from groups of flavonoids and glycosides identified in stevia leaves, which is of great importance for the nutritional composition of stevia [7]. Therefore, GSP can be added into various food products as an additive for technological purposes. For instance, it can be used for the replacement of sweetness in fruit juices in full correspondence with the growing demand for reducing added sugars in food products. Moreover, the addition of GSP increases the amount of antioxidant bioactive compounds in the juices [8,9,10,11].

The foodstuff traditional processing technologies mainly rely on heat treatments [12,13]. Apart from these traditional processes, there are other innovative processing preservation techniques based on the application of non-conventional treatments without the use of chemical preservatives and with a significant preservation of nutritional characteristics [14,15,16,17].

One of these innovative processing techniques is ultrasound technology [18,19,20]. Sonication is considered to have a minimal detrimental effect on the major quality attributes of fruit juices. Sonication can achieve relative microorganism and enzyme inactivation, as well as color and flavor preservation of some fruit juices. Moreover, this technology can improve the extractability of high-added value compounds, and can be combined with mild temperatures, thus decreasing the processing time, reducing energy costs, and improving the shelf life [16,21]. In addition, sonication is highly applicable in the juice processing industry, since it is environmentally friendly. Moreover, it can preserve and/or even enhance the nutritional and bioactive value of the product [22,23].

Therefore, the aim of this study was to evaluate the sonication processing conditions (temperature, amplitude, and time) in order to enhance the antioxidant compounds (phenolic profile) in strawberry juices sweetened with green stevia powder. Moreover, the impact of ultrasound on steviol glycosides will be also evaluated.

## 2. Results and Discussion

### 2.1. Phenolic Profile and Content/Antioxidant Capacity of Juice Samples

Table 1 contains the results obtained for the total antioxidant compounds and capacity of the analyzed samples. As can be seen in the table, significant differences (*p* ≤ 0.0001) were observed for the total phenolic compounds and flavonoid content among the different samples: control sample juices without added green stevia powder, (CS), juices with added green stevia powder (JGSP), and sonicated juice samples with added green stevia powder (SJGSP).

The analysis of variance (ANOVA) showed significant differences between the total phenolic content (TPC), total flavonoids (TF), and total antioxidant capacity (TAC) between the CS, JGSP, and SJGSP samples, obtaining the lowest total phenolic content (927.34 mg GAE L^−1^) for CS. This is attributed to the high content of phenolic compounds contained in stevia green powder (≈53.96 mg g^−1^) [2,5,24,25,26,27].

Significant differences were also observed in the TPC of SJGSP according to the sonication processing conditions, varying from 962.69 mg GAE L^−1^ (C1) to 1185.59 mg GAE L^−1^ (C18). From the obtained results, an increase (23%) in the TPC of the sample treated with the largest probe diameter (PD, 22 mm), amplitude (A) of 100%, and 25-min treatment was found compared to the juice sample treated with a PD of 7 mm and A of 50% for 15 min. When the results of SJGSP were compared to JGSP, an increase of 18% in the TPC of sonicated juice sample C18 was observed compared to the sample without sonication.

Regarding total flavonoids, the lowest values (619.94 to 652.33 mg GAE L^−1^) were obtained for the JGSP (C1–C5 and C10–C11). Although stevia green powder contains an important amount of flavonoid compounds [27,28] perhaps some flavonoid compounds from strawberry juices could be degraded under some specific sonication conditions. For instance, the ANOVA analysis proved a valid statistical difference (*p* ≤ 0.0001) between the sonicated samples combined with different ultrasonic amplitudes (50%, 75%, and 100%), probe diameters (7 mm and 22 mm) and sonication time (15 min, 20 min, and 25 min), observing values ranging from 619.94 mg L^−1^ (C1) to 791.49 mg L^−1^ (C18).

From the results obtained for the total phenolic compounds and flavonoids in sonicated juice samples, it can be concluded that the sonication treatment using higher probe diameters, amplitudes and sonication times can increase their content. For instance, the potential of ultrasound to enhance polyphenols’ extractability without decreasing their content has been previously reported [11,15,17,29]. During sonication cavitation phenomena, a breakdown or decomposition of certain substances (e.g., water) can occur, thus resulting in the formation of hydrogen ions (H^+^), free radicals (O^−^, OH^−^, HO_2_^−^), and hydrogen peroxide (H_2_O_2_). The formed OH^−^ radicals can alter polyphenolic compounds by increasing their hydroxylation degree [30,31].

In order to verify the results obtained with spectrophotometric methods and study in depth the how sonication treatment can affect individual phenolic compounds, the HPLC phenolic profile was evaluated. Flavones such as luteolin and apigenin were identified and quantified in JGSP and SJGSP, while they were not found in CS. Moreover, flavonols such as myricetin, kaempferol, and quercetin were identified in all of the samples, except for C1 to C3, where kaempferol was not detected. Similar results were obtained in research by Hohnová et al. [25], suggesting that kaempherol was not identified in sonicated samples, which may be a result of the lower ultrasound power used. According to the HPLC phenolic profile, in total, five flavonoids were identified in samples with added green stevia powder (JGSP and SJGSP), which is in agreement with results by other authors, who also observed an important content of flavonoid compounds in stevia leaves, in particular, rutin, myricetin, quercetin, luteolin, apigenin, and kaempferol [26,28].

Overall, from all of the analyzed flavonoid compounds, myricetin was the most abundant one followed by luteolin, quercetin, and apigenin, while the lowest values were found for kaempferol. Moreover, when the effect of the sonication processing parameters was evaluated, it was found that the values of these compounds significantly differed according to the probe diameter, amplitude, and sonication time, observing, in general, the highest values for the samples treated using higher probe diameters, amplitudes, and sonication time (C18), while the lowest values were found for sample C1, which used the lowest PD, A, and sonication time. For example, luteolin content ranged from 38.66 μg g^−1^ (sample C1) to 130.75 μg g^−1^ (sample C18), quercetin content ranged from 18.78 (sample C1) to 150.71 μg g^−1^ (sample C18), apigenin content ranged from 14.89 (sample C1) to 138.71 μg g^−1^ (sample C18), myricetin content ranged from 223.33 (sample C1) to 960.34 μg g^−1^ (sample C18), and kaempferol was not detected in juice samples C1, C2, and C3, while the highest value was 23.98 μg g^−1^ (C18) (Table 2).

For instance, during the sonication of the juice samples in treatment C18, luteolin, quercetin, apigenin, myricetin, and kaempferol contents increased by factors of 2.9, 5.3, 7.2, 3.2 and 5.2, respectively, compared to the juice samples with added stevia powder (without sonication of juice, JGSP). In this line, several authors have emphasized the exceptional efficiency of ultrasound at improving the extractability of polyphenolic compounds in a wide range of matrices, from wine to different types of fruit juices [5,29]. In addition, Hohnová et al. [26] studied various extraction techniques of stevia flavonoids (PFE, Soxhlet extraction and ultrasonic extraction) found the highest content of flavonoid compounds in the samples treated with high intensity ultrasound in shorter time.

When the interaction between the factors analyzed was evaluated, time and probe diameter (T × PD) and time and amplitude (T × A) did not show any significant interaction for all of the analyzed flavonoid compounds. However, the interaction of the probe diameter and amplitude (PD × A) factors was statistically significant for the content of quercetin and apigenin, while the contents of myricetin, luteolin, and kaempferol were not significantly influenced by the mentioned interaction. The interaction T × PD × A significantly affected (*p* < 0.0001) the flavonoid compounds in strawberry juice samples.

On the other hand, the content of the three of most common phenolic acids in stevia leaves was analyzed (Table 3). The average content of individual phenolic acids in juice samples with added stevia powder was as follows: pyrogallol (573.24 μg g^−1^), 4-methylcatechol (17.83 μg g^−1^), and 4-methoxybenzoic acid (21.08 μg g^−1^). Regarding phenolic acids, the predominant acid was pyrogallol, followed by 4-methoxybenzoic acid and 4-methylcatechol. These results in agreement with the findings reported by Kim et al. [2], who also observed that these compounds were predominant in stevia leaves.

The content of pyrogallol in sonicated juice samples ranged from 482.94 μg g^−1^ (A 75%, PD 7 mm; 15 min—C4) to 1999.31 μg g^−1^ (A 100%, PD 22 mm, 25 min—C18), while the 4-methylcatechol content ranged from 18.97 μg g^−1^ (A 50%, PD 7 mm, 15 min—C1) to 439.59 μg g^−1^ (C18), and the 4-methoxybenzoic acid ranged from 10.91 μg g^−1^ (C1) to 296.61 μg g^−1^ (A 75%, PD 22 mm, 25 min—C15).

In the sonicated juice samples with A 100%, PD 22 mm treated for 15 min (C16), 20 min (C17), and 25 min (C18), the decrease of 4-methoxybenzoic acid content was observed compared to juice samples treated with A 75%, PD 22 mm during 25 min (C15). Specifically, high levels of ultrasound amplitude (100%) and a larger probe diameter (22 mm) for all of the treatment times (15 min, 20 min, and 25 min) caused a reduction (−11%) of the 4-methoxybenzoic acid content in relation to the maximum obtained value in the samples in treatment C15.

During the sonication of the strawberry juice samples (CS), it was observed that the interaction of the factors time and probe diameter (T × PD) and time–amplitude (T × A) did not have a significant impact on the pyrogallol, 4-methylcatechol, and 4-methoxybenzoic acid content. A significant interaction (*p* ≤ 0.0001) of probe diameter and amplitude (PD × A) for the 4-methylcatechol and 4-methoxybenzoic acid content was found, while the pyrogallol content was not significantly affected. On the other hand, the combination of all three varied ultrasound factors (T × PD × A) significantly (*p* ≤ 0.0001) influenced the analyzed phenolic acid content.

Moreover, the total antioxidant capacity of the samples was evaluated using the ABTS free radical method (Table 1). As expected, juice samples with added green stevia powder presented the highest antioxidant values compared to the samples without stevia powder, due to the high content of phenolic phytochemicals such as flavonoids and phenolic acids found in green stevia powders [2,3,28]. For instance, the available literature data confirms the strong antioxidant activity of stevia at the level of the whole plant, especially different extracts prepared from dried stevia leaf [3,24,27,32,33].

When the effect of sonication was evaluated, it was found that the lower ultrasonic amplitude levels (50% and 75%) and smaller PD (7 mm) over 15 min, 20 min, and 25 min (samples C1 to C5) did not significantly contribute to the increase of antioxidant activity of the samples, as the flavonoids and phenolic acids were not enhanced. For instance, the highest antioxidant capacity was obtained for the C16 to C18 samples, which also contained the highest amounts of total phenols, flavonoids, and glycosides. The ultrasonic parameters (PD), sonication time (T), and amplitude (A) in common interaction significantly affected the increased total phenolic and flavonoid content as well as the antioxidant capacity of the juice samples with added green stevia powder treated with high-power ultrasound. For the interaction of factors, there were no statistical significant differences between time and probe diameter (T × PD) and time and amplitude (T × A), while significant interactions were observed. (*p* ≤ 0.0001) between PD and A (PD × A) and all three ultrasound variables: time, PD, and A (T × PD × A).

### 2.2. The Content of Steviol Glycosides in Juice Samples

Table 4 shows the results of the predominant steviol glycosides in stevia leaves (stevioside and rebaudioside A (Reb A). The average content of stevioside and Reb A in juice samples with added green stevia powder was 70.42 mg g^−1^ and 18.49 mg g^−1^, respectively; these results are in agreement with those found in the available literature, which indicated stevioside and Reb A contents ranging from 4% to 20% [34,35] and from 2% to 4% [36], respectively. Specifically, when the content of stevioside and Reb A almost equal greater sensory quality, a more pleasant sweet taste to consumers is increased, and bitterness is greatly reduced [37,38].

The Reb A and steviol glycoside contents in the sonicated samples were significantly higher in the ultrasound-treated samples compared to the untreated samples; their content was ≈2 times and 61% higher. Based on the obtained results, it is important to emphasize the effectiveness of sonication to promote the release of steviol glycosides, but it is important to take into account the optimal variation of key ultrasound variables. For example, lower A (50%), smaller PD (7 mm), and treatment times of 15 min and 20 min (C1 and C2) did not increase the stevioside content compared to the untreated samples. However, longer times (25 min) under the same ultrasonic conditions of A (50%) and PD (7 mm) significantly contributed to the increase (5%) of the stevioside content (C3) compared to the samples without sonication. The Reb A content significantly increased (≈7%) under lower ultrasonic conditions A (50%) and smaller PD (7 mm) during 20 min compared to the samples without sonication. A significant increase of stevioside and Reb A content was determined for samples C3 to C10.

Lower stevioside contents (~28%) were determined in the sonicated juice samples under the following conditions: a lower level of ultrasonic A (50%), 15 min, and a PD of 22 mm (C10) compared to juice sample C9 with an A of 100%, PD of 7 mm, and 25-min treatment time. In addition, under the above-mentioned ultrasonic conditions, a lower Reb A content by up to 24% in the juice sample was found in sample C10 compared to the sample C9. A possible explanation for the lower stevioside and Reb A content in juice samples with combined larger PD (22 mm), lower A levels, and shorter sonication time could be attributed to a reduced effectiveness in extracting certain chemical compounds [39]. The significance analysis of the interaction among the different ultrasonic variables confirmed the above variations in the results of the stevioside and Reb A content in juice samples. According to the statistical analysis, the interaction between time and amplitude (T × A), time and probe diameter (T × PD), and probe diameter and amplitude (PD × A) did not significantly affect the change in the stevioside content. For the Reb A content, the interactions between time and probe diameter (T × PD), and probe diameter and amplitude (PD × A) did not have significant impact on the modifications in the content of this compound, whereas the interaction of the time and amplitude (T × A) factors showed slightly significant differences (*p* = 0.02). The interaction of all three investigated ultrasonic factors (T × PD × A) had a significant (*p* < 0.001) influence on the change in the stevioside and Reb A contents.

## 3. Material and Methods

### 3.1. Plant Material

Stevia plants (*Stevia rebaudiana* Bertoni) were cultivated in experimental field conditions. The seeds were sown in the greenhouse. After 50 days, when the basic plant habitus was formed, seedlings were transferred into polyethylene pots. Then, they were grown in an open space during mid-May. The spacing between plants was 80 × 40 cm, thereby achieving a plant density of 3.1 plants/m^2^. The soil preparation in the open, before planting, including basic fertilization with N:P:K fertilizer in a ratio of 5:20:30 and mulching with black polyethylene foil. Irrigation was not applied during the cultivation of the plants, and water availability depended on the climatic conditions. From the plants, only green, healthy leaves were collected in the late September. Stevia leaves were dried in a convection dryer (INKO ST40T, Inkolab, Zagreb, Croatia) in an elemental layer at 55 °C for 2 h. Afterwards, dried leaves were milled in a laboratory mill (IKA MF-10, IKA, Staufen, Germany). The prepared green stevia powder was stored and used for the further described experiments.

### 3.2. Sample Preparation

For research purposes, strawberry juice was used as the targeted matrix. Juice was prepared from strawberry fruit cultivars ‘Joly’, ‘Alba’, and ‘Albion’ (2:1:1) and divided into three main groups: (i) control samples (juices without green stevia powder, CS), (ii) strawberry juices with green stevia powder (JGSP), and (iii) sonicated juices with green stevia powder (SJGSP). Juices with green stevia powder (JGSP) were prepared as follows: 500 milligrams (±0.0001) of green stevia powder were added to 150 mL of strawberry juice, samples were allowed to stand for 24 h at room temperature with intermittent shaking, and finally, the samples were filtered. While, for the third group (SJGSP), 500 mg (±0.0001) of green stevia powder were added to 150 mL of strawberry juice, and the sonication process was carried out using an ultrasound probe system (UP 400S, Hielscher Company, Teltow, Germany). The power ultrasound device consisted of a transducer for converting electric signals into ultrasonic waves using a probe with a specific diameter. The maximal nominal output power of the device was 400 W, and the ultrasonic frequency was 24 kHz. The experimental design of sonication is shown in Table 5. During the sonication, the following parameters were combined and tested: amplitude (50%, 75%, and 100%), probe diameter (7 mm and 22 mm) and time (15 min, 20 min, and 25 min). The temperature change was measured with a laser thermometer (Raytek – MiniTemp FS, Burlington, NJ, USA) at intervals of 30 s. The maximum recorded temperature during the sonication of juice samples for 30 min, with a probe diameter of 7 mm and amplitude of 100%, was 63.9 °C, while in the same operational conditions with a probe diameter of 22 mm, the maximum temperature was 80.9 °C.

### 3.3. Determination of Total Phenolic and Flavonoid Content and Phenolic and Flavonoid Profile by HPLC Analysis

The total phenols content was assessed according to Shukla et al. [24] with some modifications. The measurements were carried out as follows: 1 mL of the prepared extract and 1 mL of Folin–Ciocalteu reagent diluted with distilled water (1:2) were added into a volumetric flask of 50 mL and allowed to stand for 3 min. Additionally, 3 mL of a saturated sodium carbonate solution was added; then, the flask was filled to the mark with distilled water and allowed to stand for 3 h at room temperature with intermittent shaking. The absorbance of the blue color generated after the reaction was measured spectrophotometrically (UV 1650 PC, Shimadzu, Buckinghamshire, UK) at 750 nm using distilled water as a blank. The total flavonoid content was obtained according to the method previously reported [24] with some modifications: 1 mL of the prepared extracts, 1 mL of 20% HCl (*v*/*v*), and 0.5 mL of formaldehyde were added into a volumetric flask of 25 mL. Then, the samples were blown with nitrogen and allowed to stand for 24 h at room temperature. Afterwards, the same Folin–Ciocalteu reagent used for the determination of the total phenolic compounds was employed. The absorbance was measured spectrophotometrically at 750 nm with distilled water as a blank. The gallic acid (y = 0.0009x + 0.0141; R^2^ = 0.997) was used as an external standard, and the concentration of total phenols and flavonoids was expressed as mg GAE L^−1^.

For the determination of the individual phenolic compounds, primarily flavones (apigenin, luteolin), flavonols (myricetin, kaempherol, quercetin), and phenolic acids (pyrogallic acid, 4-methylcatechol, and 4-methoxybenzoic acid) with different HPLC conditions were used. All of the standards of mentioned flavonoids (99% purity) were obtained from Sigma Aldrich (Darmstadt, Germany). Twenty-five milliliters of strawberry juice were evaporated on a rotary evaporator R-215 (Büchi, Switzerland) according to the following conditions: 135 rpm, a water bath temperature of 55 °C, and pressure of 55 mbar. The samples were evaporated to a final volume of less than 1 mL. Five milliliters of methanol (HPLC grade) were added into the evaporated samples. Then, the samples were filtered through Nylon filters of 0.45 μm (Macherey-Nagel, Düren, Germany) in a vial. The determination of individual flavones and flavonols compounds was obtained according to Hohnová et al. [26]. The Varian 920 LC with Galaxie software (Varian, Sydney, Australia) was used in combination with a multiple UV wavelength detector equipped with an autoinjector, autosampler, and quaternary pump. Separation was done on a Nucleosil C-18, 5 μm (250 × 4.6 mm I.D.) with a Nucleosil C-18, guard column, 5 μm (10 × 4.6 mm I.D.).

To achieve the separation of flavonoid compounds, HPLC operating conditions incorporated a gradient mobile phase: A—acetonitrile (HPLC grade), pH 2.5; B—water (HPLC grade), pH 2.3 adjusted with acetic acid (HPLC grade). The following gradient elution was used: elution started with 5% A and 95% B, which remained the linear gradient for 20 min. From 20 to 25 min, elution was kept isocratic with 60% A and 40% B. Then, a linear gradient was used to reach 100% A until 35 min. The operating conditions were the following: flow rate, 0.7 mL min^−1^; column temperature, 20 °C; and a 20-μL injection volume of the standards and extract samples. The UV detector was used to monitor at 360 nm with a reference signal set at 367 nm. The final flavones and flavonols results were recalculated according to the linear equation of standards: apigenin, y = 0.9773x + 2.644 (R^2^ = 0.9841); luteolin, y = 1.0642x + 6.086 (R^2^ = 0.9881); myricetin, y = 1.0027x + 7.3067 (R^2^ = 0.9899); kaempherol, y = 0.9883x + 0.7299 (R^2^ = 0.999); quercetin, y = 1.0005x + 1.6218 (R^2^ = 0.9998); and expressed as μg g^−1^. The determination of phenolic acids was obtained according to Kim et al. [2]. Separation was performed on a 5-μm Nucleosil C-18 (250 × 4.6 mm I.D.) with a 5-μm Nucleosil C-18 guard column (10 × 4.6 mm I.D.). In order to separate the phenolic acids, the HPLC operating conditions involved a gradient mobile phase: A—50 mM of sodium buffer:methanol (93:7, *v*/*v*); B—methanol solution (70%, *v*/*v*). The binary gradient elution was utilized for the separation of flavonoid compounds: 0–15 min linear from 100% to 70% A; 15–45 min linear from 70% to 65% A; 45–65 min linear from 65% to 60% A; 65–70 min linear from 60% to 50% A; 70–95 min linear from 50% to 0% A; 95–100 min linear from 0% to 100% A; and 100–105 min isocratic at 100% A. The operating conditions were the following: a flow rate 0.7 mL min^−1^, a column temperature of 20 °C, and an injection volume of 20 μl of the standards and the extract samples. The UV detector was set to monitor at 280 nm. The phenolic acids results were recalculated according to the linear equation of standards expressed as μg g^−1^: pyrogallic acid y = 0.4946x + 22.014 (R^2^ = 0.9992), 4-methylcatechol y = 0.7382x − 0.15 (R^2^ = 0.9979), 4-methoxybenzoic acid y = 0.5878x + 2.9651 (R^2^ = 0.9985).

### 3.4. Determination of Steviol Glycosides

In order to determine the steviol glycosides, the separation was done on a 5-μm Nucleosil C-18 (250 × 4.6 mm I.D.) with a 5-μm Nucleosil C-18 guard column, (10 × 4.6 mm I.D.). To optimize the separation of aqueous stevia extracts, HPLC operating conditions incorporated a gradient mobile phase as follows: A—water (HPLC grade), pH 5 adjusted with acetic acid; B—acetonitrile (HPLC grade), pH 5. The following gradient elution was applied for the separation of stevioside and rebaudioside A: elution started with 80% A and 20% B, which remained the linear gradient for 20 min. From 20 to 25 min, elution was maintained isocratic with 20% A and 80% B. Then, a linear gradient was applied to reach 80% A and 20% B until 28 min. The operating conditions were the following: flow rate, 1.0 mL min^−1^; column temperature, 20 °C; and injection volume, 10 μL of the standards and the extract samples. The UV detector was adjusted to monitor at 210 nm with a reference signal adjusted at 360 nm. For quantification purposes, stevioside (98% purity) and rebaudioside A (96% purity) standards were purchased from Sigma-Aldrich (Darmstadt, Germany). The results were recalculated according to the linear equation of standards and expressed as mg g^−1^: stevioside, y = 28.779x + 0.2951 (R^2^ = 0.9996); Reb A, y = 22.822x + 0.5927 (R^2^ = 0.9987).

### 3.5. Determination of Antioxidant Capacity by ABTS Method of Strawberry Juice Samples

The antioxidant activity of the juice samples was determined by the ABTS method. Trolox (6-hydroxy-2,5,7,8-tetramethylchroman-2-carboxylic acid; Sigma-Aldrich) was used as an antioxidant standard. Trolox (2.5 mM) was prepared in ethanol (80%) to be used as a stock standard. ABTS, 2, 2′-azinobis (3-ethylbenzothiazoline-6-sulfonic acid), and potassium persulfate were supplied from Sigma-Aldrich. The experiments were done according to Miller et al. [40]. ABTS (7 mM) and potassium persulfate (140 mM) were dissolved in distilled water. These two solutions, i.e., 88 μL of the prepared potassium persulfate (140 mM) and 5 mL of the prepared ABTS solution, were mixed, and the mixture was incubated under darkness at room temperature for 16 h before using it in order to produce ABTS radicals (ABTS^•+^). On the day of the quantification, 1% ABTS solution (1 mL of ABTS in a volumetric flask of 100 mL filled with 96% ethanol up to the mark) was prepared. The absorbance of the ABTS radical solution was quantified at 734 nm and set to absorbance of 0.70 ± 0.02. One hundred and sixty microliters of the extracts obtained were placed in the cuvette, mixed with 2 mL of 1% ABTS^•+^, and the absorbance at 734 nm was measured.

### 3.6. Statistical Analysis

For statistical analysis, a generalized linear model with included repetitions, the extraction method as a categorical predictor, and all of the interactions of time, amplitude, and the probe diameter during ultrasonic treatment were used. All of the treatments, i.e., control sample (CS), juices with green stevia powder (JGSP) and sonication (SJGSP) were performed in triplicate. For the analysis procedure, the PROC GLM in SAS software package (SAS, Cary, NC, USA), version 9.3. was used. The obtained data were analyzed using an analysis of the variance (ANOVA), while the differences among means were tested using LSD test, where *p* = 1% was considered statistically significant.

## 4. Conclusions

Sonication processing conditions had an important influence on the antioxidant compounds and capacity, observing an enhanced content after using the highest probe diameter (22 mm), amplitude (100%), and sonication time (25 min). For instance, it not had only an important impact on the total compounds, but also on the flavones, flavonols, phenolic acids, and steviol glycosides profile. The antioxidant capacity of strawberry juice samples directly correlates with the content of studied bioactive compounds and the applied treatment method. By comparing the effectiveness of different processing methods on the content of the bioactive compounds, it can be concluded that the selection of sonication optimal conditions could strongly contribute to the enhancement of antioxidant components and the nutritional quality of strawberry juice with added green stevia. The present study highlights the possibility of sonication application as an effective tool for the preservation and isolation of thermolabile bioactive compounds, but also emphasizes that green stevia powder is a rich source of valuable phytochemicals and justifies its addition to enhance the bioactive and nutritional quality of the final product.

## Figures and Tables

**Table 1:** The content of total phenols (mg GAE L^−1^), flavonoids (mg GAE L^−1^), and antioxidant capacity (mmol TE L^−1^) of the juice samples.

Treatments	TPC	TFC	Antioxidant Capacity
	*p* ≤ 0.0001	*p* ≤ 0.0001	*p* ≤ 0.0001
**CS**			
	927.34 ^m^ ± 55.62	659.03 ^l^ ± 39.60	3.774 ^m^ ± 28.71
**JGSP**			
	1003.23 ± 13.66 ^d^	685.17 ± 40.67^j^	3.84 ± 9.64 ^g^
**SJGSP**			
C1	962.69 ± 0.60 ^l^	619.94 ± 0.84 ^r^	3.81 ± 0.79 ^k,l^
C2	967.93 ± 0.19 ^k^	624.04 ± 0.08 ^q^	3.82 ± 0.01 ^j^
C3	976.98 ± 2.72 ^i^	632.62 ± 2.27 ^o^	3.82 ± 0.37 ^i^
C4	971.30 ± 0.50 ^j^	627.94 ± 0.43 ^p^	3.82 ± 1.33 ^j^
C5	979.55 ± 1.28 ^h^	641.93 ± 4.19 ^n^	3.83 ± 2.63 ^h^
C6	987.12 ± 1.24 ^f^	697.66 ± 1.08 ^i^	3.84 ± 0.55 ^f^
C7	976.38 ± 1.15 ^i^	687.50 ± 2.41 ^j^	3.85 ± 1.14 ^e^
C8	980.29 ± 0.49 ^g,h^	724.66 ± 0.71 ^f^	3.86 ± 0.005 ^d^
C9	991.53 ± 0.65 ^e^	742.03 ± 0.07 ^e^	3.81 ± 2.05 ^j,k^
C10	967.54 ± 0.50 ^k^	649.42 ± 1.35 ^m^	3.82 ± 1.50 ^j^
C11	977.54 ± 0.92 ^i^	652.33 ± 0.57 ^m^	3.81 ± 0.36 ^l^
C12	986.38 ± 0.80 ^f^	665.31 ± 0.64 ^k^	3.76 ± 1.17 ^o^
C13	971.46 ± 0.29 ^j^	705.16 ± 0.51 ^h^	3.75 ± 0.86 ^p^
C14	982.03 ± 1.21 ^g^	763.45 ± 1.07 ^d^	3.76 ± 0.87 ^o^
C15	992.08 ± 0.005 ^e^	782.52 ± 0.57 ^b^	3.77 ± 0.38 ^n^
C16	1067.80 ± 0.45 ^c^	720.76 ± 0.92 ^g^	3.86 ± 2.45 ^c^
C17	1123.56 ± 1.08 ^b^	771.05 ± 0.80 ^c^	3.88 ± 0.47 ^b^
C18	1185.59 ± 0.195 ^a^	791.49 ± 1.15 ^a^	3.89 ± 1.15 ^a^
Interaction of Sonication Factors			
T × PD	NS	NS	NS
T × A	NS	NS	NS
PD × A	***	**	***
T × PD × A	***	***	***

CS—control sample (strawberry juice); JGSP—strawberry juices with green stevia powder; SJGSP—sonicated juices with green stevia powder; TPC—total phenol content; TFC—total flavonoid content, T × PD—time and probe diameter, T × A—time and amplitude, PD × A—probe diameter and amplitude, T × PD × A—time, probe diameter and amplitude. NS—not significant, ** *p* ≤ 0.001, *** *p* ≤ 0.0001. Different uppercase letters in the same column indicate significant differences between treatments, representing ^a^ the highest value.

**Table 2:** The content of flavones and flavonols (μg g^−1^) in juice samples.

Treatments	Luteolin	Apigenin	Myricetin	Kaempherol	Quercetin
	*p* ≤ 0.0001	*p* ≤ 0.0001	*p* ≤ 0.0001	*p* ≤ 0.0001	*p* ≤ 0.0001
**CS**					
	ND	ND	1.93 ± 0.01 ^t^	3.26 ± 0.01 ^o^	2.51 ± 0.03 ^s^
**JGSP**					
	44.45 ± 0.02 ^o^	19.16 ± 0.02 ^q^	297.13 ± 0.01 ^q^	4.58 ± 0.01 ^m^	28.43 ± 0.02 ^o^
**SJGSP**					
C1	38.66 ± 0.45 ^r^	14.89 ± 0.05 ^s^	223.33 ± 0.02 ^s^	ND ^p^	18.78 ± 0.05 ^r^
C2	42.42 ± 0.68 ^q^	16.94 ± 0.01 ^r^	285.16 ± 0.24 ^r^	ND ^p^	20.77 ± 0.01 ^q^
C3	44.29 ± 0.01 ^p^	42.53 ± 0.01 ^o^	343.63 ± 0.05 ^o^	ND ^p^	32.77 ± 0.30 ^k^
C4	44.29 ± 0.05 ^p^	38.43 ± 0.63 ^p^	330.04 ± 0.60 ^p^	2.73 ± 0.01 ^n^	24.77 ± 0.05 ^p^
C5	64.97 ± 0.02 ^m^	64.01 ± 0.05 ^n^	488.61 ± 0.01 ^m^	7.79 ± 0.02 ^k^	29.77 ± 0.01 ^m^
C6	68.73 ± 0.02 ^l^	70.15 ± 0.01 ^k^	501.93 ± 0.02 ^l^	12.85 ± 0.01 ^g^	32.76 ± 0.02 ^l^
C7	73.42 ± 0.05 ^j^	82.43 ± 0.01 ^h^	404.84 ± 0.05 ^n^	11.86 ± 0.05 ^h^	28.76 ± 0.01 ^n^
C8	99.74 ± 0.45 ^g^	84.78 ± 0.23 ^g^	566.40 ± 0.01 ^k^	14.97 ± 0.65 ^e^	36.76 ± 0.05 ^i^
C9	105.37 ± 0.02 ^f^	164.29 ± 0.01 ^a^	624.25 ± 0.05 ^h^	20.07 ± 0.01 ^c^	99.73 ± 0.23 ^f^
C10	52.75 ± 0.02 ^n^	67.08 ± 0.02 ^l^	589.34 ± 0.02 ^j^	4.76 ± 0.02 ^l^	35.76 ± 0.02 ^j^
C11	70.61 ± 0.05 ^k^	77.32 ± 0.05 ^j^	590.34 ± 0.42 ^i^	11.84 ± 0.01 ^i^	70.75 ± 0.05 ^h^
C12	121.35 ± 0.01 ^d^	103.92 ± 0.01 ^f^	755.89 ± 0.01 ^f^	14.88 ± 0.01 ^f^	98.73 ± 0.01 ^g^
C13	88.46 ± 0.01 ^i^	66.06 ± 0.01 ^m^	641.20 ± 0.01 ^g^	8.80 ± 0.05 ^j^	114.72 ± 0.01 ^e^
C14	111.95 ± 0.01 ^e^	121.31 ± 0.16 ^e^	842.66 ± 0.05 ^c^	12.85 ± 0.01 ^g^	115.72 ± 0.05 ^d^
C15	128.87 ± 0.02 ^b^	132.57 ± 0.01 ^c^	869.59 ± 0.01 ^b^	15.87 ± 0.02 ^d^	146.71 ± 0.01 ^b^
C16	94.09 ± 0.02 ^h^	79.36 ± 0.05 ^i^	768.86 ± 0.01 ^e^	11.84 ± 0.02 ^i^	115.72 ± 0.01 ^d^
C17	125.11 ± 0.01 ^c^	122.33 ± 0.01 ^d^	811.74 ± 0.01 ^d^	22.97 ± 0.01 ^b^	143.71 ± 0.01 ^c^
C18	130.75 ± 0.05 ^a^	138.71 ± 0.01 ^b^	960.34 ± 0.02 ^a^	23.98 ± 0.01 ^a^	150.71 ± 0.01 ^a^
Interaction of Sonication Factors					
T × PD	NS	NS	NS	NS	NS
T × A	NS	NS	NS	NS	NS
PD × A	NS	**	NS	*	**
T × PD × A	***	***	***	***	***

CS—control sample (strawberry juice); JGSP—strawberry juices with green stevia powder; SJGSP—sonicated juices with green stevia powder; ND—not determined. T × PD—time and probe diameter, T × A—time and amplitude, PD × A—probe diameter and amplitude, T × PD × A—time, probe diameter and amplitude. NS—not significant, * 0.01 ≤ *p* ≤ 0.05 ** *p* ≤ 0.001, *** *p* ≤ 0.0001. Different uppercase letters in the same column indicate significant differences between treatments, representing ^a^ the highest value.

**Table 3:** The content of phenolic acids (μg g^−1^) in juice samples.

Treatments	Pyrogallic Acid	4-methylcatechol	4-methoxybenzoic Acid
	*p* ≤ 0.0001	*p* ≤ 0.0001	*p* ≤ 0.0001
**JGSP**			
	573.24 ± 1.17 ^o^	17.83 ± 1.56 ^m^	21.08 ± 0.63 ^l^
**SJGSP**			
C1	483.95 ± 1.01 ^q,r^	18.97 ± 0.68 ^m^	10.91 ± 0.01 ^o^
C2	486.98 ± 0.01 ^p,q^	27.77 ± 0.01 ^k^	14.73 ± 0.55 ^n^
C3	487.99 ± 1.02 ^p^	27.43 ± 0.34 ^k^	16.91 ± 0.55 ^m^
C4	482.94 ± 0.01 ^r^	23.03 ± 0.68 ^l^	11.99 ± 0.01 ^o^
C5	940.37 ± 1.52 ^m^	29.13 ± 1.36 ^k^	14.18 ± 1.10 ^n^
C6	996.48 ± 2.02 ^l^	33.19 ± 1.36 ^j^	22.36 ± 0.55 ^l^
C7	642.66 ± 0.01 ^n^	22.35 ± 1.36 ^l^	17.45 ± 0.01 ^m^
C8	1095.52 ± 1.01 ^k^	38.61 ± 0.01 ^i^	25.63 ± 0.55 ^k^
C9	1313.91 ± 0.01 ^i^	43.35 ± 0.68 ^h^	28.35 ± 0.01 ^j^
C10	1297.60 ± 2.04 ^j^	19.64 ± 1.35 ^m^	63.54 ± 1.70 ^i^
C11	1514.07 ± 0.01 ^g^	90.76 ± 0.68 ^g^	112.88 ± 1.70 ^h^
C12	1780.95 ± 2.02 ^d^	136.14 ± 1.36 ^f^	186.03 ± 0.01 ^g^
C13	1469.59 ± 0.01 ^h^	134.11 ± 0.68 ^f^	215.80 ± 0.85 ^f^
C14	1867.89 ± 2.03 ^c^	214.04 ± 0.68 ^e^	238.77 ± 0.01 ^e^
C15	1943.71 ± 1.01 ^b^	274.32 ± 1.36 ^c^	296.61 ± 1.70 ^c^
C16	1524.18 ± 2.02 ^f^	252.64 ± 1.35 ^d^	265.99 ± 1.70 ^d^
C17	1688.96 ± 3.03 ^e^	350.86 ± 0.68 ^b^	273.17 ± 2.40 ^b^
C18	1999.31 ± 2.02 ^a^	439.59 ± 1.36 ^a^	276.57 ± 0.01 ^a^
Interaction of Sonication Factors			
T × PD	NS	NS	NS
T × A	NS	NS	NS
PD × A	NS	***	***
T × PD × A	***	***	***

JGSP—strawberry juices with green stevia powder; SJGSP—sonicated juices with green stevia powder. T × PD—time and probe diameter, T × A—time and amplitude, PD × A—probe diameter and amplitude, T × PD × A—time, probe diameter, and amplitude. NS—not significant, *** *p* ≤ 0.0001. Different uppercase letters in the same column indicate significant differences between treatments, representing ^a^ the highest value.

**Table 4:** The content of steviol glycosides (mg g^−1^) in juice samples.

Treatments	Stevioside	Rebaudioside A
	*p* ≤ 0.0001	*p* ≤ 0.0001
**JGSP**		
	70.42 ± 0.53 ^I,h^	18.49 ± 2.63 ^I,h^
**SJGSP**		
C1	65.73 ± 0.01 ^k^	17.84 ± 0.66 ^i^
C2	68.86 ± 0.02 ^I,j^	19.81 ± 0.01 ^g,h^
C3	73.94 ± 5.25 ^g^	20.47 ± 0.66 ^g^
C4	66.77 ± 0.01 ^j,k^	19.81 ± 0.01 ^g,h^
C5	69.89 ± 0.01 ^i^	21.13 ± 0.01 ^f,g^
C6	74.06 ± 0.02 ^g^	22.44 ± 0.02 ^e,f^
C7	84.48 ± 0.01 ^d,e^	23.10 ± 0.66 ^e^
C8	90.74 ± 0.01 ^b^	27.70 ± 0.01 ^c^
C9	93.35 ± 0.52 ^b^	30.99 ± 0.66 ^b^
C10	73.02 ± 0.01 ^g,h^	25.07 ± 0.02 ^d^
C11	74.59 ± 0.53 ^g^	25.07 ± 0.01 ^d^
C12	86.57 ± 1.04 ^c,d^	27.05 ± 0.66 ^c^
C13	81.36 ± 0.01 ^f^	21.13 ± 0.01 ^f,g^
C14	82.40 ± 0.01 ^e,f^	23.10 ± 0.66 ^e^
C15	86.05 ± 0.52 ^c,d^	27.05 ± 0.66 ^c^
C16	87.62 ± 1.05 ^c^	26.38 ± 0.01 ^c,d^
C17	92.31 ± 0.53 ^b^	30.99 ± 0.66 ^b^
C18	115.24 ± 0.52 ^a^	39.53 ± 0.01 ^a^
Interaction of Sonication Factors		
T × PD	NS	NS
T × A	NS	*
PD × A	NS	NS
T × PD × A	***	***

JGSP—strawberry juices with green stevia powder; SJGSP—sonicated juices with green stevia powder. T × PD—time and probe diameter, T × A—time and amplitude, PD × A—probe diameter and amplitude, T × PD × A—time, probe diameter, and amplitude. NS—not significant, * 0.01 ≤ *p* ≤ 0.05, *** *p* ≤ 0.0001. Different uppercase letters in the same column indicate significant differences between treatments, representing ^a^ the highest value.

**Table 5:** Experimental conditions for sonication treatments (SJGSP).

Matrix	V (mL)	T (°C)	A (%)	PD (mm)	Time (min)	Treatments
SJ	150	21.5	50	7	15	C1
					20	C2
					25	C3
SJ	150	21.5	75	7	15	C4
					20	C5
					25	C6
SJ	150	21.5	100	7	15	C7
					20	C8
					25	C9
SJ	150	21.5	50	22	15	C10
					20	C11
					25	C12
SJ	150	21.5	75	22	15	C13
					20	C14
					25	C15
SJ	150	21.5	100	22	15	C16
					20	C17
					25	C18

V—matrix volume; T—initial matrix temperature; A—amplitude; PD—probe diameter; SJ: sonicated juice.
